# Combined Effects of Bee Venom Acupuncture and Morphine on Oxaliplatin-Induced Neuropathic Pain in Mice

**DOI:** 10.3390/toxins8020033

**Published:** 2016-01-22

**Authors:** Woojin Kim, Min Joon Kim, Donghyun Go, Byung-Il Min, Heung Sik Na, Sun Kwang Kim

**Affiliations:** 1 Department of Physiology, College of Korean Medicine, Kyung Hee University, 26 Kyungheedae-ro, Dongdamoon-gu, Seoul 02447, Korea; thasnow@gmail.com; 2 Department of East-West Medicine, Graduate School, Kyung Hee University, 26 Kyungheedae-ro, Dongdamoon-gu, Seoul 02447, Korea; junesnest@naver.com (M.J.K.); drum2r@daum.net (D.G.); 3 Yeongju Municipal Hospital, 697 Jangan-ro, Anjeong-myeon, Gyeongsangbuk-do, Yeongju-si 36051, Korea; mbi@khu.ac.kr; 4 Department of Physiology, College of Medicine, Korea University, Anam-dong 5-ga, Seongbuk-gu, Seoul 02842, Korea; hsna@korea.ac.kr

**Keywords:** bee venom acupuncture, chemotherapy induced neuropathic pain, morphine, oxaliplatin

## Abstract

Oxaliplatin, a chemotherapeutic drug for colorectal cancer, induces severe peripheral neuropathy. Bee venom acupuncture (BVA) has been used to attenuate pain, and its effect is known to be mediated by spinal noradrenergic and serotonergic receptors. Morphine is a well-known opioid used to treat different types of pain. Here, we investigated whether treatment with a combination of these two agents has an additive effect on oxaliplatin-induced neuropathic pain in mice. To assess cold and mechanical allodynia, acetone and von Frey filament tests were used, respectively. Significant allodynia signs were observed three days after an oxaliplatin injection (6 mg/kg, i.p.). BVA (0.25, 1, and 2.5 mg/kg, s.c., ST36) or morphine (0.5, 2, and 5 mg/kg, i.p.) alone showed dose-dependent anti-allodynic effects. The combination of BVA and morphine at intermediate doses showed a greater and longer effect than either BVA or morphine alone at the highest dose. Intrathecal pretreatment with the opioidergic (naloxone, 20 μg) or 5-HT_3_ (MDL-72222, 15 μg) receptor antagonist, but not with α_2_-adrenergic (idazoxan, 10 μg) receptor antagonist, blocked this additive effect. Therefore, we suggest that the combination effect of BVA and morphine is mediated by spinal opioidergic and 5-HT_3_ receptors and this combination has a robust and enduring analgesic action against oxaliplatin-induced neuropathic pain.

## 1. Introduction

Oxaliplatin is a third-generation platinum-based chemotherapy drug commonly used for colorectal cancer [1], which is the third most common cancer in men and the second most common cancer in women worldwide [2]. It is widely used as there are no nephrotoxicity and ototoxicity symptoms, unlike after the use of other platinum based drugs, such as cisplatin and carboplatin [3,4]. However, it causes peripheral neuropathy characterized by dysesthesias of the hands and feet, which is a major dose-limiting side effect. Even a single administration of oxaliplatin can evoke this abnormal sensation [5,6]. About 90% of oxaliplatin-treated patients rapidly develop significant pain without motor dysfunction during or shortly after a single infusion, peaking at the first 24–48 h [7]. Efforts are underway to determine an efficacious treatment, but currently, an optimal treatment without major side effects is not available [8,9]. Therefore, an effort to determine novel therapeutic options is urgently needed.

Bee venom acupuncture (BVA) is a treatment method that involves injecting diluted bee venom into acupoints, and it has been used in Korean medicine to treat various diseases, such as cervical disc protrusion [10], progressive muscle atrophy [11], idiopathic Parkinson’s disease [12], and cancer [13] in humans and animals. In addition, it has been traditionally used to alleviate pain, like arthritis pain [14] and musculoskeletal pain [15], and the results of a clinical trial have suggested that BVA can be effective in chemotherapy induced peripheral neuropathy (CIPN) [16]. Recent experimental studies performed in our laboratory have demonstrated that it effectively attenuates peripheral neuropathic pain induced by a single injection of oxaliplatin [17,18]. Unlike electroacupuncture, in which the analgesic effect is mediated via opioids as well as other various receptors [19,20], the analgesic actions of BVA have been reported to be mediated by the descending pain inhibitory system, involving spinal noradrenergic and serotonergic receptors, but not opioidergic receptors [17,21,22].

Morphine is a well-known analgesic drug and its effect is most likely mediated via opioid receptors [23]. However, whether it can efficaciously attenuate neuropathic pain is still a controversial issue. Previous studies have reported that neuropathic pain might be resistant or less responsive to opioids than nociceptive pain and higher dosages of opioidergic agents are required to evoke the same pain-relieving effects [24,25,26]. However, the American Society of Clinical Oncology still does not provide recommendations regarding the use of opioids for the treatment of CIPN [27]. Nonetheless, some articles have reported that morphine effectively attenuates neuropathic pain [28], and furthermore, that morphine can significantly suppress the neuropathic pain induced by a single injection of oxaliplatin in rats [29], suggesting that morphine might be effective in CIPN. These results let us speculate that the combination treatment of BVA and morphine might have an additive effect, as the analgesic effect of BVA is mediated by spinal noradrenergic and/or serotonergic receptors, and the effect of morphine is mediated by opioidergic receptors.

In this article, firstly, we investigated whether and to what extent BVA or morphine administered alone at different doses can attenuate oxaliplatin-induced cold and mechanical allodynia in mice, and secondly, we assessed whether the combination treatment of these two mechanistically distinct analgesic treatment strategies can improve analgesic efficacy compared to the use of one agent alone. Finally, we determined which spinal receptors were involved in mediating the effects of the combined BVA and morphine treatment.

## 2. Results

### 2.1. Induction of Cold and Mechanical Allodynia by a Single Intraperitoneal Administration of Oxaliplatin in Mice

**Figure 1:**
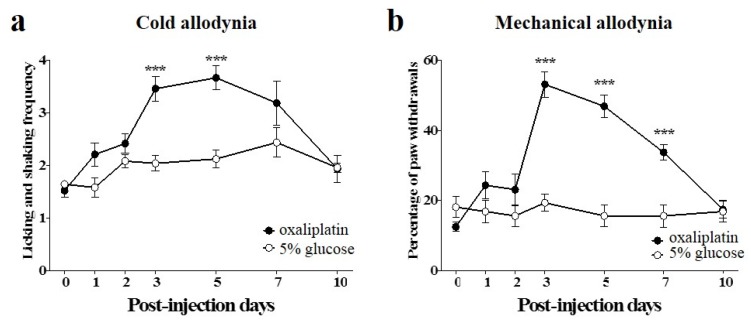
Time courses of cold and mechanical allodynia induced by a single injection of oxaliplatin in mice. (**a**,**b**) Behavioral tests for cold and mechanical allodynia were performed before (day point zero) and after the administration of oxaliplatin (6 mg/kg, i.p.). 5% glucose was used as control and was injected intraperitoneally. Cold and mechanical allodynia were assessed by the acetone and von Frey filament tests, respectively. Data are presented as the mean ± S.E.M. for 8 mice; *** *p* < 0.001 *vs.* 5% glucose, by unpaired *t*-test.

Single administration of oxaliplatin (6 mg/kg, i.p.) significantly increased the frequencies of licking and shaking of the hind paw in response to cold acetone stimuli (10 μL), from 3 to 5 days after the injection, compared to the vehicle injected group (Figure 1a). Also, single administration of oxaliplatin significantly increased the withdrawal responses of the hind paw to von Frey filament (0.4 g bending force) application (expressed as percentage of paw withdrawals) at day 3, and they were maintained up to day 7 (Figure 1b). We interpreted these results as a sign of cold and mechanical allodynia as they indicate that mice in the experimental group developed significant hypersensitivity to cold and mechanical stimuli. Treatment with 5% glucose as control, had no effect on cold and mechanical sensitivity (Figure 1). These results are in accordance with other articles, where a single injection of oxaliplatin induced allodynia in rodents [29,30].

### 2.2. Time Course of Dose-Dependent Effects of BVA or Morphine on Oxaliplatin-Induced Cold and Mechanical Allodynia in Mice

**Figure 2:**
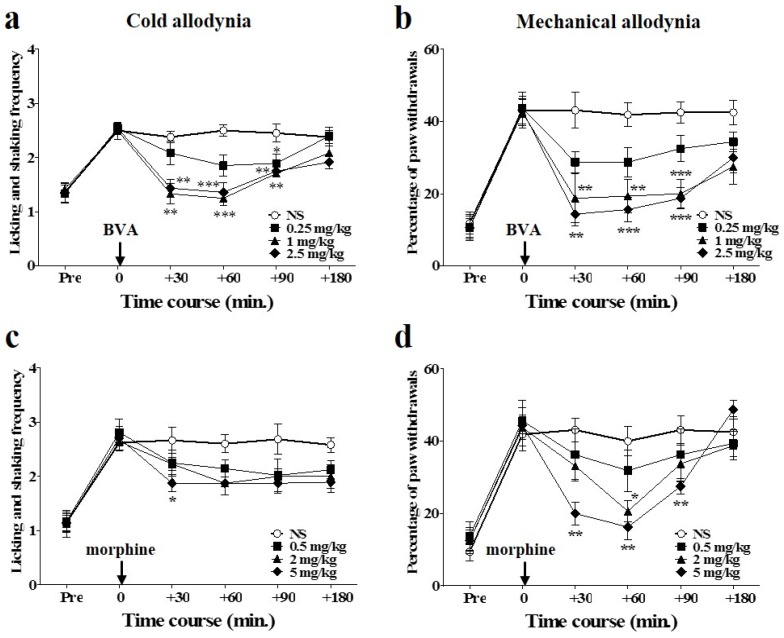
Time course of dose-dependent effects of BVA or morphine on oxaliplatin-induced cold and mechanical allodynia in mice. (**a**,**b**) Time course and dose response of BVA for cold and mechanical allodynia. Normal saline (NS, control) and three different doses of BVA (0.25, 1, and 2.5 mg/kg) were subcutaneously administered at ST36 acupoint (*n* = 8/group). (**c**,**d**) Time course and dose response of morphine for cold and mechanical allodynia. NS and three different doses of morphine (0.5, 2, and 5 mg/kg) were injected intraperitoneally (*n* = 8/group). “Pre” refers to the assessment made before the injection of oxaliplatin. Data are presented as the mean ± S.E.M.; * *p* < 0.05, ** *p* < 0.01, *** *p* < 0.001 *vs.* NS by Bonferroni post-test after one-way ANOVA.

The anti-allodynic effects of different doses of BVA (s.c., ST36 acupoint) or morphine (i.p.) are shown in Figure 2. All assessments were conducted three days after the injection of oxaliplatin, when cold and mechanical allodynia were significantly induced. To observe the time course of the effects, evaluations were conducted at time point zero (before the administration of BVA or morphine), and at 30, 60, 90, and 180 min after the initiation of the treatments. The effects of BVA on cold and mechanical allodynia are shown in Figure 2a,b. The medium and highest doses of BVA (1 and 2.5 mg/kg) exhibited similar relieving effects, lasting until 90 min after the injection, for both cold and mechanical allodynia, although the highest dose of BVA appeared to have a slightly greater suppressive effect. The effect of a lower dose (0.25 mg/kg) of BVA was less significant compared to that in the 1 or 2.5 mg/kg treated groups. Among the mice treated with three different doses of BVA, some of the mice in the 2.5 mg/kg treated group only showed swelling at the injected area.

A single injection of morphine also produced dose-dependent inhibitory effects on oxaliplatin-induced cold and mechanical allodynia (Figure 2c,d). In the acetone test, only the high dose (5 mg/kg) of morphine had a significant anti-allodynic effect at 30 min after the treatment, and the 0.5 and 2 mg/kg doses had no significant effect compared to that in the control group. In the von Frey filament test, the analgesic effect of the medium dose of morphine (2 mg/kg) was observable only at 60 min after the treatment, and the effect of the 5 mg/kg dose lasted longer (90 min) than the effect of the 2 mg/kg dose. Neither of the effects of these remained until 180 min. The lowest dose (0.5 mg/kg) of morphine had no significant effect on both cold and mechanical allodynia.

### 2.3. Time Course of Combined Effects of BVA and Morphine on Oxaliplatin-Induced Cold and Mechanical Allodynia in Mice

**Figure 3:**
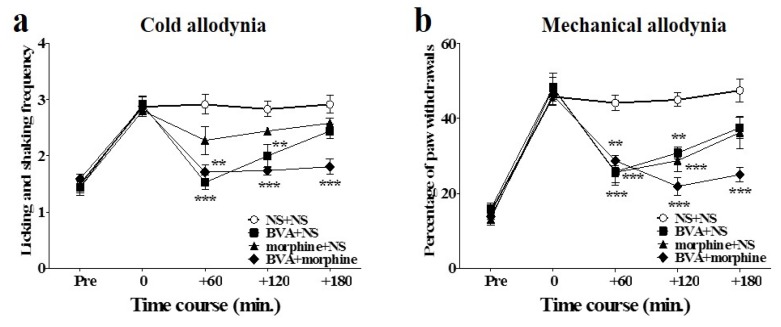
Time course of the effect of combined administration of BVA and morphine on oxaliplatin-induced cold and mechanical allodynia in mice. BVA (1 mg/kg, s.c., ST36) and morphine (2 mg/kg, i.p.) were administered simultaneously. Effect on cold (*n* = 6–8/group) (**a**) and mechanical (*n* = 6–8/group) (**b**) allodynia were assessed using the acetone and von Frey filament test, respectively. NS was administered subcutaneously at ST36, when used with morphine, and administered intraperitoneally when used with BVA. Data are presented as the mean ± S.E.M.; ** *p* < 0.01, *** *p* < 0.001 *vs.* control (NS + NS); by Bonferroni post-test after one-way ANOVA.

The combined effects of BVA and morphine on oxaliplatin-induced cold and mechanical allodynia in mice are shown in Figure 3. Intermediate doses of BVA (1 mg/kg, s.c., ST36) and morphine (2 mg/kg, i.p.) were administered simultaneously. The anti-allodynic effect of the combined administration of BVA and morphine was stronger and lasted longer than the effect of the combination of BVA or morphine with NS (*i.e.*, NS + NS, BVA + NS, and morphine + NS) on both cold and mechanical allodynia. In the acetone test, BVA with NS had a strong effect at the beginning (60 min), but its analgesic effect disappeared at 180 min after administration. Morphine with NS did not have any significant analgesic effect. Combination of BVA and morphine had a longer lasting effect than that in the other groups (Figure 3a). In the von Frey filament test, both morphine with NS and BVA with NS had significant effects that lasted until 120 min after the treatment. However, these effects were less significant than the effect of the combination treatment, which lasted until 180 min (Figure 3b).

### 2.4. Involvement of Spinal Opioidergic and 5-HT_3_ Receptors but not of α_2_-Adrenergic Receptors in the Combination Effect of BVA and Morphine on Oxaliplatin-Induced Allodynia in Mice

**Figure 4:**
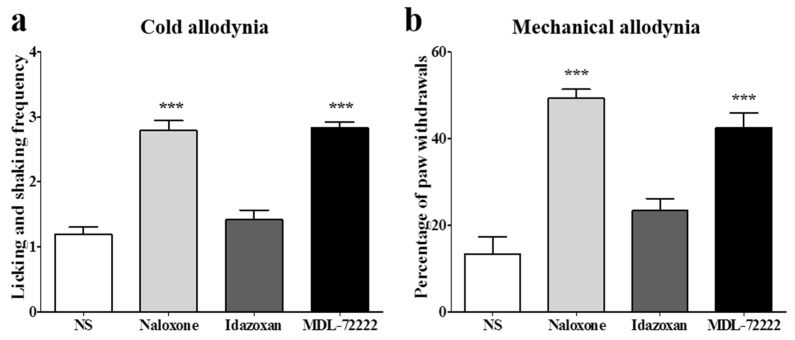
Involvement of spinal opioidergic and 5-HT_3_, but not of α_2_-adrenergic receptors, in the combination effect of BVA and morphine on oxaliplatin-induced allodynia in mice. The behavioral tests for cold (**a**) and mechanical (**b**) allodynia were performed. Twenty min after the pretreatment with antagonists, BVA (1 mg/kg) and morphine (2 mg/kg) were administered. Assessment was made 60 min after the BVA and morphine treatment. NS (i.t., *n* = 6). Naloxone (20 μg, i.t., *n* = 7). Idazoxan (10 μg, i.t., *n* = 6). MDL-72222 (15 μg, i.t., *n* = 6). Data are presented as the mean ± S.E.M.; *** *p* < 0.001 *vs.* NS; by Bonferroni post-test after one-way ANOVA.

To clarify the analgesic mechanism of combined BVA and morphine administration at the spinal cord level, opioidergic, noradrenergic, and serotonergic antagonists were injected intrathecally 20 min before the treatment. Naloxone (opioid antagonist, 20 μg), idazoxan (α_2_-adrenergic antagonist, 10 μg), or MDL-72222 (5-HT_3_ receptor antagonist, 15 μg) was administered in a volume of 5 μL, and the same volume of NS was used for control. Idazoxan, similar to the NS treated group, did not block the anti-allodynic effect of the combined administration of BVA and morphine, showing that spinal α_2_-adrenergic receptors are not involved in the analgesic effect of BVA and morphine. In contrast, the naloxone pretreated group or the MDL-72222 pretreated group significantly blocked the analgesic effect of BVA and morphine, indicating that spinal opioidergic and 5-HT_3_ receptors may play a crucial role in the suppressive effect of BVA and morphine on oxaliplatin-induced cold and mechanical allodynia in mice (Figure 4). In each group, mice showed elevated cold and mechanical allodynia signs before the administration of each antagonist (cold-licking and shaking frequency: NS 3.05 ± 0.43; naloxone 3.02 ± 0.29; idazoxan 2.83 ± 0.45; MDL-72222 2.72 ± 0.20, mechanical-percentage of paw withdrawals: NS 46.66 ± 6.05; naloxone 46.42 ± 5.56; idazoxan 49.16 ± 3.76; MDL-72222 46.66 ± 6.83).

## 3. Discussion

Peripheral neuropathy occurring soon after the administration of oxaliplatin is a serious side effect that limits the use of the drug. Anticonvulsants and serotonin-norepinephrine reuptake inhibitors are recommended as first-line treatments, but even these drugs can cause side effects, such as sedation, dizziness, and cardiac complications. Thus, there is a need for better therapeutic options [31,32]. 

BVA is a therapeutic method generally used in Korea. It has been reported to effectively attenuate neuropathic pain in different animal models via noradrenergic and/or serotonergic pathways [17,18,33], and the results of a clinical trial have demonstrated that BVA can be efficacious in CIPN [16]. Morphine, although polemics exist [24,25,26], has been demonstrated to significantly alleviate neuropathic pain [28,34] and oxaliplatin-induced peripheral neuropathy [29]. These results let us hypothesize that BVA, along with morphine, might be effective for oxaliplatin-induced cold and mechanical allodynia.

In this article, firstly, we observed the time course and dose response of the separate BVA and morphine effect on oxaliplatin-induced allodynia in mice. BVA doses of 1 and 2.5 mg/kg had a statistically significant effect on both cold and mechanical allodynia. However, a high dose of BVA induced a side effect (*i.e.*, swelling at the injected area) in a subset of mice as observed in our previous study [17], suggesting that a BVA dose of 1 mg/kg is the most effective dose without any side effects, as reported in another article [21]. In our experiments, 0.25 mg/kg dose of BVA had a less significant effect than 1 or 2.5 mg/kg dose of BVA, unlike the previous data, where the lowest dose of BVA (0.25 mg/kg) had a greater effect than higher doses (1 or 2.5 mg/kg) [17]. This discrepancy may be due to the differences in the area of behavioral test (hind paw *vs.* tail), location of acupoint (ST36 *vs.* GV3), and animal strain (mice *vs.* rats). In addition, we found that morphine dose-dependently attenuated the allodynia elicited by oxaliplatin in mice. Its analgesic effects were greater on mechanical allodynia than on cold allodynia. In the acetone test for cold allodynia, only the 5 mg/kg treated group showed a significant decrease in allodynia at 30 min after the treatment. In the von Frey filament test, both the 2 and 5 mg/kg doses significantly attenuated allodynia, but these effects were not maintained until 180 min after the injection. Although no behavioral oddity such as sedation was observed in the 5 mg/kg group in these experiments, an article reported that a statistically significant reduction in spontaneous locomotor activity was observed in 5 mg/kg of morphine injected mice [35].

Secondly, we demonstrated that the combination treatment of medium doses of BVA (1 mg/kg) and morphine (2 mg/kg) can markedly reduce cold and mechanical allodynia induced by a single injection of oxaliplatin in mice. The combination treatment of BVA and morphine had an analgesic effect that lasted longer (180 min) than the effect of the highest doses of BVA or morphine (2.5 mg/kg or 5 mg/kg, respectively). Furthermore, no side effect such as swelling was observed unlike in mice injected with the highest dose of BVA. Thus, we propose that the combination of medium doses of BVA and morphine can be an effective treatment method, without any side effects, in oxaliplatin-induced neuropathic pain.

Thirdly, in the following study, which was conducted to clarify the mechanism of the analgesic effect of the combined treatment of BVA and morphine, opioid and 5-HT_3_ receptors but not α_2_-adrenergic receptors were found to mediate the effect at the spinal level. These results were somewhat unexpected because, hitherto, only a limited number of studies have concentrated on the role of serotonergic receptors [22], compared to the numerous articles that have focused on α_2_-adrenergic receptors [21,36,37,38,39], and the analgesic pathways of BVA in various types of pain. However, experiments conducted in our laboratory demonstrated that intrathecal injections of noradrenergic antagonists partially blocked the analgesic effects of BVA on oxaliplatin-induced cold allodynia in rats [17], whereas serotonergic antagonists completely blocked the effects [18]. Thus, it can be suggested that the serotonergic analgesic system rather than the noradrenergic system plays an important role in the effect of BVA, at least on oxaliplatin-induced neuropathic pain.

As mentioned in the “Introduction” section, BVA and morphine have been known to modulate two distinct analgesic pathways: BVA via noradrenergic and/or serotonergic receptors [22], and morphine via opioidergic receptors [23]. However, in our results, pre-treatment with naloxone or MDL-72222 completely, not partially, blocked the analgesic effect of the combined treatment of BVA and morphine. Although we could not understand all the details of the process, these results let us speculate that other pathways may be involved in the analgesic effect of the combination treatment of BVA and morphine. Actually, complex interrelationships between opioid and serotonergic systems at the spinal or supraspinal level have been suggested. For example, it has been suggested that enkephalinergic dorsal horn neurons express 5-HT_3_ receptors, and that at least some part of the analgesia produced by 5-HT at the spinal level is due to 5-HT_3_ receptors located on the intrinsic neurons in the dorsal horn [40,41]. Some articles supported this suggestion by demonstrating that the analgesic effect of intrathecally administered 5-HT_3_ receptor agonist 2-methylserotonin was attenuated by the opioid antagonist naloxone [42,43]. Furthermore, microinjection of morphine into the periaqueductal gray has been found to evoke the release of serotonin from the spinal cord [44].

In conclusion, intermediate doses of BVA (1 mg/kg) and morphine (2 mg/kg), when administered simultaneously, significantly attenuated cold and mechanical allodynia induced by a single administration of oxaliplatin in mice. This combination of BVA and morphine at intermediate doses had a greater and longer effect than either BVA or morphine alone at the highest dose (*i.e.*, 2.5 mg/kg or 5 mg/kg, respectively). This analgesic effect was significantly blocked by intrathecally administered naloxone and MDL-72222, but not by idazoxan, demonstrating that opioidergic and 5-HT_3_ receptors, but not α_2_-adrenergic receptors, mediate the effect at the spinal level.

## 4. Experimental Section

### 4.1. Animals

Male C57BL/6 mice (6–8 weeks old) were purchased from Daehan Biolink (Chungbuk, Korea) and they were housed in cages with water and food available *ad libitum*. The room was maintained with 12 h-light/dark cycle (a light cycle; 08:00–20:00, a dark cycle; 20:00–08:00) and kept at 23 ± 2 °C. All animals were acclimated in their cages for one week prior to any experiments. All procedures involving animals were approved by the Institutional Animal Care and Use Committee of Kyung Hee University (KHUASP(SE)-15-024).

### 4.2. Oxaliplatin Administration

Oxaliplatin (Sigma, St. Louis, MO, USA) was dissolved in a 5% glucose solution at a concentration of 2 mg/mL and was intraperitoneally (i.p.) injected at a dose of 6 mg/kg [4,45]. The same volume of 5% glucose solution was used in the control group.

### 4.3. Behavioral Test

Behavioral tests were conducted before and after oxaliplatin administration to observe whether cold and mechanical allodynia were induced in mice. For a week before the start of experiments, each mouse was habituated to handling procedures and to all testing procedures by investigators. The experimenters were blinded to oxaliplatin and any other treatments. Cold and mechanical sensitivities were measured using acetone and von Frey filaments, respectively [45]. Mice were placed on a wire mesh floor covered with a clear plastic box (12 × 8 × 6 cm), and were habituated for 30 min prior to each testing. Acetone (10 μL, Reagents Chemical Ltd., Gyonggi-do, Korea) was sprayed three times onto the plantar skin of each hind paw, and the frequencies of licking and shaking of the affected paw was counted after the acetone spray, for 30 s [46]. It is possible that acetone induces some behavioral responses in naïve mice; however, as shown in our experiments, mice that received a single injection of oxaliplatin showed a significantly increased level of response to acetone, compared to the vehicle injected control mice.

Mechanical sensitivity was measured by the von Frey filament test [47]. Mice were placed on the same wire mesh floor covered with a clear plastic box as mentioned above, and the von Frey filament (Linton Instrumentation, Norfolk, UK) with a bending force of 0.4 g was applied ten times to the mid plantar skin (avoiding the base of the tori) of each hind paw, and each application was held for 3 s [48]. The number of withdrawal responses to the von Frey filament applications from both hind paws was counted and then expressed as an overall percentage response.

### 4.4. BVA and Morphine Treatment

BV from *Apis mellifera* (Sigma) was dissolved in NS. BV was subcutaneously injected into the right Zusanli acupoint (ST36) located on the lateral side of the stifle joint adjacent to the anterior tubercle of the tibia as previously described [36,49]. Morphine hydrochloride (Myungmoon Pharm., Seoul, Korea) was diluted in NS and was injected intraperitoneally. In order to determine the effective dose of BVA or morphine, mice showing significant cold and mechanical allodynia signs were divided randomly into four groups. BVA at doses of 0.25, 1.0, and 2.5 mg/kg was injected into ST36 acupoint in a volume of 20 μL. In the control group, the same volume of NS was injected subcutaneously into the same acupoint. For morphine, doses of 0.5, 2, and 5 mg/kg were injected intraperitoneally in a volume of 0.2 mL. Control group mice received the same volume of NS, intraperitoneally.

### 4.5. Antagonist Treatment

In order to reveal the spinal analgesic mechanism of simultaneously injected BVA and morphine in oxaliplatin-administered mice, the antagonists were injected intrathecally. Opioid receptor antagonist naloxone (Sigma; 20 μg), selective α_2_-adrenergic receptor antagonist idazoxan (Sigma; 10 μg), or 5-HT_3_ antagonist MDL-72222 (Tocris, Cookson, UK, 3-tropanyl-3,5-dichlorobenzoate, 15 μg) were injected 20 min prior to the combination treatment of BVA and morphine.

Naloxone and idazoxan were dissolved in NS. MDL-72222 was dissolved in 20% dimethyl sulfoxide (DMSO). The needle of a Hamilton syringe was inserted into the subarachnoid space between lumbar vertebrae L5 and L6. A flick of the mouse’s tail provided a reliable indicator that the needle had penetrated the dura mater. The syringe was held in position for a few seconds after the injection of a volume of 5 μL. The dose of each antagonist was determined based on previously published studies showing the selective and effective antagonistic action [38,50,51,52].

### 4.6. Statistical Analysis

The data are presented as mean ± S.E.M. and were analyzed by the paired, unpaired *t*-test, or one-way ANOVA followed by the Bonferroni multiple comparison test was performed to determine the statistically significant differences among the groups. *p* < 0.05 was considered statistically significant.
